# Importance of Preserved Ratio Impaired Spirometry as a Risk Factor for Development of COPD, Also in Those Who Do Not Smoke

**DOI:** 10.1016/j.chest.2025.02.025

**Published:** 2025-03-08

**Authors:** Helena Backman, Tomi Myrberg, Linnea Hedman, Caroline Stridsman, Eva Rönmark, Anne Lindberg

**Affiliations:** a OLIN and Sunderby Research Unit, Department of Public Health and Clinical Medicine, Umeå University, Umeå, Sweden; b Department of Diagnostics and Intervention, Anesthesiology and Intensive Care Medicine (T. M.), Umeå University, Umeå, Sweden

**Keywords:** asthma, case-control studies, COPD, dyspnea, PRISm, productive cough, respiratory symptoms, smoking, spirometry

## Abstract

**Background:**

COPD is largely underdiagnosed. Active identification of cases is crucial to establish preventive measures before manifestation of clinical disease. The significance of different spirometric patterns preceding COPD, especially preserved ratio impaired spirometry (PRISm), has been highlighted but remains unclear.

**Research Question:**

Which clinical characteristics, smoking habits, and spirometric patterns, with primary focus on PRISm findings, precede the development of airway obstruction (AO)?

**Study Design and Methods:**

The OLIN COPD Study was established from 2002 through 2004. After re-examination of population-based cohorts, individuals with AO (n = 993; FEV_1_ to VC ratio < 0.70) were identified together with control participants without AO (n = 993; FEV_1_ to VC ratio ≥ 0.70). Most of these people had participated in examinations during the 1980s or 1990s, and in total, 902 cases and 819 control participants had previous clinical data. Logistic regression was performed with case status as outcome and spirometric patterns, age, sex, smoking habits, and BMI at first examination as covariates.

**Results:**

The mean (SD) person-years between first examination and inclusion in the OLIN COPD Study was 10.5 (4.0) years. At first examination, the prevalence of PRISm was higher in cases (18.6%) vs control participants (13.4%). Current smoking was more common in cases (45.1% vs 18.2%), whereas former smoking was similar (31.8% vs 34.9%). Cases reported more respiratory symptoms (78.0% vs 44.3%) than control participants. At first examination, PRISm, current smoking, and former smoking were strongly associated with becoming a case when adjusted for confounders, with adjusted OR (aOR) of 3.5, 4.1, and 1.5, respectively. When stratifying for smoking habits, aORs for PRISm in those with current smoking, former smoking, and nonsmoking status were 2.9, 3.8 and 3.7, respectively.

**Interpretation:**

In this study, PRISm was associated with transition into AO corresponding to COPD within 1 decade, independent of smoking habits and with similar strength of association among those who have never smoked, who formerly smoked, and who currently smoke.

**Take-Home Points**

**Study Question:** How are smoking habits and spirometric patterns—with the primary focus on the preserved ratio impaired spirometry (PRISm) pattern—associated with the development of airway obstruction (AO) in adults?

**Results:** PRISm findings as well as both former and current smoking were associated with future development of AO in this retrospective case-control study, and importantly, PRISm was associated with development of AO also in those who did not smoke.

**Interpretation:** Our findings warrant further studies to explore potential underlying disease mechanisms that may be associated with spirometric patterns and the development of COPD, and further knowledge on PRISm may reveal potential preventive measures against COPD, beyond the mainstay of smoking cessation.

COPD is a major health care burden worldwide and one of the leading causes of death. The estimated global prevalence is around 10%,^1^ and it is expected to increase within the coming decades.^2^ The major risk factor for COPD in medium-income to high-income countries is smoking,^1^ whereas globally, exposure to biomass combustion may be of similar importance.^3^ However, the underdiagnosis of COPD is large, and only 30% to 50% of individuals with COPD are identified by interactions with health care administration.^4^^,^^5^ The most recent updates of the Global Initiative for Chronic Obstructive Lung Disease strategy document^6^ advocate active identification of cases to reduce underdiagnosis and to identify individuals who may benefit from current therapies.

Case detection also is important for identifying individuals at risk of COPD, that is, symptomatic individuals without airflow obstruction, covered by the umbrella term *pre-COPD*.^6^ It has been shown that both preserved ratio impaired spirometry (PRISm) (normal FEV_1_ to FVC ratio, but low FEV_1_) and restrictive spirometric pattern (RSP) (normal FEV_1_ to FVC ratio, but low FVC) may develop into airflow obstruction^7^^,^^8^ and even increase mortality.^7^, ^8^, ^9^ Moreover, these spirometric patterns overlap,^10^ but whether RSP and PRISm are associated similarly with the development of COPD remains unclear. Further, to what extent these spirometric patterns precede COPD relative to known risk factors such as smoking remains to be explored.

Thus, the aim of the current study was to evaluate clinical characteristics, smoking habits, and spirometric patterns preceding the development of airway obstruction (AO), with the primary focus on PRISm. From the OLIN COPD Study, which includes individuals with and without AO identified after re-examinations of population-based cohorts from 2002 through 2004, we used retrospective data including spirometry from the first examinations of these cohorts > 1 decade earlier.

## Study Design and Methods

### Study Population

The OLIN Studies epidemiologic research program started in 1985. The first 4 population-based cohorts were recruited by postal questionnaire surveys. Cohorts I and II included age-stratified samples recruited in 1985 and 1992, respectively, whereas cohorts III and IV included random samples recruited in 1992 and 1996, respectively. In total, cohorts I through IV comprise almost 30,000 primarily White individuals, from which random and stratified samples of participants were invited to clinical examinations.

From 2002 through 2004, the previously examined individuals within cohorts I through IV were invited to re-examinations, after which all individuals with AO (FEV_1_ to vital capacity [VC] ratio < 0.70; n = 993 cases) were identified together with age-matched and sex-matched control participants without AO (FEV_1_ to VC ratio ≥ 0.70; n = 993 control participants). This study population (n = 1,986) constitute the OLIN COPD study, and the study design was described previously in detail.^11^

For each case and control participant in the OLIN COPD study, we retrospectively identified data from their first clinical examination (Table 1), which for cohorts I through III was carried out during the 1980s or 1990s. In total, we could include 902 cases and 819 control participants (e-Fig 1). All studies were approved by the Ethical Review Board of Umeå University (Identifier: 2004-045M) and followed the tenets of the Declaration of Helsinki. The reporting of this study conforms to the Strengthening the Reporting of Observational Studies in Epidemiology statement.^12^

**Table 1:** Clinical Characteristics and Comorbidities at the First Examination Among Future Cases and Control Participants

Variable	Cases (n = 902)	Control Participants (n = 819)	*P* Value
Demographics			
Age, y	54.9 (11.4)	56.3 (11.3)	**.016**
Female sex	368 (44.9%)	407 (45.1%)	.937
BMI, kg/m^2^	25.4 (3.7)	25.9 (3.6)	**.008**
Smoking habits			
Nonsmoking	208 (23.1%)	384 (46.9%)	**< .001**
Former smoking	287 (31.8%)	286 (34.9%)	.173
Current smoking	407 (45.1%)	149 (18.2%)	**< .001**
Respiratory symptoms, diagnoses, and medications			
Productive cough 3 mo/y for at least 2 y^a^	387 (42.9%)	158 (19.3%)	**< .001**
Dyspnea (mMRC dyspnea scale ≥ 2)	198 (22.0%)	75 (9.2%)	**< .001**
Wheeze last 12 mo	607 (67.3%)	264 (32.2%)	**< .001**
Any respiratory symptoms	704 (78.0%)	363 (44.3%)	**< .001**
Physician diagnosis			
Asthma ever	202 (22.4%)	62 (7.6%)	**< 0.001**
Chronic bronchitis or emphysema	153 (17.0%)	43 (5.3%)	**< .001**
Airway medication for OAD last 12 mo	229 (25.4%)	63 (7.7%)	**< .001**
Use of ICS last 12 mo	96 (10.6%)	23 (2.8%)	**< .001**
Spirometric pattern			
Normal lung function	324 (35.9%)	680 (83.0%)	**< .001**
Obstructive spirometry	410 (45.5%)	29 (3.5%)	**< .001**
PRISm	168 (18.6%)	110 (13.4%)	**.003**
Comorbidity			
Ischemic heart disease	116 (12.9%)	69 (8.4%)	**.003**
Early life events			
Maternal smoking during pregnancy	37 (4.1%)	34 (4.2%)	.978
Passive smoking at home during childhood	497 (55.1%)	407 (49.7%)	.064
Asthma before school age	47 (5.2%)	15 (1.8%)	**< .001**
Severe airway or lung infection before school age	252 (28.0%)	228 (27.9%)	.953

Data are presented as No. (%) or mean (SD) unless otherwise indicated. Significant *P* values appear in boldface. ICS = inhaled corticosteroid; mMRC = modified Medical Research Council; OAD = obstructive airway disease; PRISm = preserved ratio impaired spirometry.

a Chronic bronchitis.

### Spirometry

All spirometry tests were performed using the same set of dry volume spirometers, the Mijnhardt Vicatest 5, following the guidelines for American Thoracic Society at the time when the study was conducted.^13^^,^^14^ VC was defined as the highest of FVC, slow vital capacity, or both. Bronchodilator tests with salbutamol (4.0 × 0.2 mg Ventoline Discus [GlaxoSmithKline]) were performed among individuals with FEV_1_ to VC ratio of < 0.70 or FEV_1_ of < 80% predicted. The OLIN reference values were used.^15^

### Spirometric Patterns at First Examination (Before Bronchodilator Administration)

Spirometric patterns at first examination (before bronchodilator administration) were as follows: AO, FEV_1_ to VC ratio < 0.70; normal lung function (NLF), FEV_1_ to VC ratio ≥ 0.70 and FEV_1_ ≥ 80% predicted; and PRISm, FEV_1_ to VC ratio ≥ 0.70 and FEV_1_ < 80% predicted.

### Clinical Characteristics at First Examination

Besides height, weight, and BMI clinical characteristics were collected by structured interview questionnaires that have been used previously in several national and international surveys.^16^, ^17^, ^18^

### Definitions of Smoking Habits and Any Respiratory Symptom at First Examination

Smoking habits were defined as follows: nonsmoking, < 1 cigarette/d for 1 year; former smoking, stopped smoking at least 12 months previously; and current smoking, currently smoking or stopped smoking during the previous 12 months. Any respiratory symptom was defined as at least one of (1) chronic productive cough 3 mo/y, (2) any wheeze in the last 12 months, or (3) dyspnea (modified Medical Research Council dyspnea scale, ≥ 2).

### Statistical Analysis

The Statistical Package for the Social Sciences software version 29 (SPSS, Inc.) was used for statistical analyses. Descriptive statistics were calculated and compared between groups using the χ^2^ test, *t* test, or analysis of variance as appropriate, using *P* < .05 as the threshold for statistical significance. Logistic regression analysis was used to calculate ORs with 95% CIs as measures of associations between covariates at first examination and becoming included as a case (control participants as reference), both unadjusted and adjusted by including spirometric patterns, age, sex, smoking habits, BMI, and original cohort (I-III) at first examination as covariates in the models. A separate model including inhaled corticosteroid use at first examination as an additional covariate was also constructed. The choice of covariates was based on previous evidence and clinical experience. The Hosmer-Lemeshow goodness-of-fit test was used for model evaluation, and the Wald test was used for significance of individual covariate estimates. Collinearity between model covariates was examined by the variance inflation factor, and all results yielded a variance inflation factor of < 1.5. Interactions between PRISm and the other covariates were evaluated in separate models. The logistic regression models were constructed by including all cases and control participants, as well as by stratifying for sex and smoking habits at first examination. In the stratified adjusted regression analyses, no adjustment was made for the variable used for stratification.

### Additional Analyses Including RSP

In additional analyses, we also took RSP (FEV_1_ to VC ratio ≥ 0.70 and FVC < 80% predicted) into account, and thus the following mutually exclusive groups were defined for spirometric patterns before bronchodilator administration at first examination: AO, FEV_1_ to VC ratio < 0.70; NLF, FEV_1_ to VC ratio ≥ 0.70 and FEV_1_ ≥ 80% predicted and FVC ≥ 80% predicted; PRISm only, FEV_1_ to VC ratio ≥ 0.70, FEV_1_ < 80% predicted, and FVC ≥ 80% predicted; RSP only, FEV_1_ to VC ratio ≥ 0.70, FVC < 80% predicted, FEV_1_ ≥ 80% predicted; and PRISm plus RSP, FEV_1_ to VC ratio ≥ 0.70, FEV_1_ < 80% predicted, and FVC < 80% predicted.

### Sensitivity Analysis Based on Cases Defined by Spirometry After Bronchodilator Administration

The main analyses were repeated based on cases defined by having an FEV_1_ to VC ratio < 0.70 in the examinations done in 2002 through 2004 using the highest values of spirometry before and after bronchodilator administration, which resulted in 662 cases and 819 control participants being included.

## Results

### Characteristics by Spirometric Pattern at First Examination Among Cases and Control Participants

Overall, a mean (SD) of 10.5 (4.0) years elapsed between the first examination and inclusion in the OLIN COPD study for both cases and control participants. At first examination, cases showed a higher prevalence of current smoking, respiratory symptoms, physician-diagnosed obstructive airway disease, ischemic heart disease, and report of having asthma before school age than control participants (Table 1).

**Figure 1:**
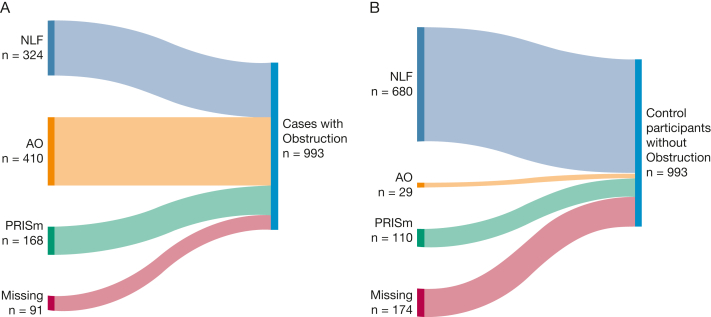
A, B, Illustrations showing distributions of spirometric patterns among cases (A) and control participants (B): NLF, AO, and PRISm or missing at first examination. AO = airway obstruction; NLF = normal lung function; PRISm = preserved ratio impaired spirometry.

Among cases, n = 324 (36%; mean age, 53 years) had NLF, n = 410 (45%; mean age, 57 years) had AO, and n =168 (19%; mean age, 56 years) had PRISm at first examination (Fig 1A, Table 2). Among control participants, n = 680 (83%; mean age, 56 years) had NLF, n = 29 (4%; mean age, 62 years) had AO, and n = 110 (13%; mean age, 59 years) had PRISm at first examination (Fig 1B, Table 2). Future cases had more respiratory symptoms, more diagnoses, and more treatments at first examination and demonstrated a more rapid decline in FEV_1_ during follow-up compared with control participants, regardless of having NLF, obstruction, or PRISm at first examination (Table 2). Among cases, individuals with obstruction and PRISm showed a similarly increased prevalence of respiratory symptoms compared with those with NLF, whereas individuals with PRISm tended to be most symptomatic among control participants (Table 2). Early life events were rather equally common among cases and control participants with the exception of asthma before school age, which was most common in cases, especially among those with obstruction already at first examination (Table 1, Table 2).

**Table 2:** Basic Characteristics at First Examination by Spirometric Groups Among Future Cases and Control Participants

Variable	NLF^a^		Obstruction^b^		PRISm^c^	
	Cases (n =324)	Control Participants (n = 680)	Cases(n = 410)	Control Participants (n = 29)	Cases (n = 168)	Control Participants (n = 110)
Demographics						
Age, y	52.6 (11.5)	55.6 (11.5)	56.5 (11.4)	62.3 (11.2)	55.7 (10.5)	59.0 (9.0)
BMI, kg/m^2^	25.4 (3.6)	25.8 (3.4)	25.1 (3.4)	27.1 (3.8)	26.3 (4.6)	26.4 (4.4)
Female sex	145 (44.8%)	297 (43.7%)	168 (41.0%)	12 (41.4%)	94 (56.0%)	59 (53.6%)
Smoking habits						
Nonsmoking	80 (24.7%)	321 (47.2%)	81 (19.8%)	11 (37.9%)	47 (28.0%)	52 (47.3%)
Former smoking	93 (28.7%)	237 (34.9%)	137 (33.4%)	12 (41.4%)	57 (33.9%)	37 (33.6%)
Current smoking	151 (46.6%)	122 (17.9%)	192 (46.8%)	6 (20.7%)	64 (38.1%)	21 (19.1%)
Respiratory symptoms, diagnoses, medication, and FEV_1_ decline						
Productive cough 3 mo/y for at least 2 y^d^	118 (36.4%)	113 (16.6%)	186 (45.4%)	9 (31.0%)	83 (49.4%)	36 (32.7%)
Dyspnea (mMRC dyspnea scale ≥ 2)	52 (16.0%)	54 (7.9%)	103 (25.1%)	3 (10.3%)	43 (25.6%)	18 (16.4%)
Wheeze last 12 mo	193 (59.6%)	189 (27.8%)	295 (72.0%)	12 (41.4%)	119 (70.8%)	63 (57.3%)
Any respiratory symptoms	233 (71.9%)	276 (40.6%)	335 (81.7%)	17 (58.6%)	136 (81.0%)	70 (63.6%)
Physician diagnosis						
Asthma ever	46 (14.2%)	43 (6.3%)	115 (28.0%)	4 (13.8%)	41 (24.4%)	15 (13.6%)
Chronic bronchitis or emphysema	47 (14.5%)	36 (5.3%)	76 (18.5%)	0 (0%)	30 (17.9%)	7 (6.4%)
Airway medication for OAD last 12 mo	50 (15.4%)	45 (6.6%)	130 (31.7%)	3 (10.3%)	49 (29.2%)	15 (13.6%)
Use of ICS last 12 mo	14 (4.3%)	14 (2.1%)	63 (15.4%)	3 (10.3%)	19 (11.3%)	6 (5.5%)
Annual FEV_1_ decline, mL	50.2 (27.8)	34.6 (25.2)	36.5 (34.7)	15.2 (31.7)	40.4 (26.5)	16.4 (27.1)
Comorbidity						
Ischemic heart disease	41 (12.7%)	48 (7.1%)	53 (12.9%)	5 (17.2%)	22 (13.1%)	16 (14.5%)
Early life events						
Maternal smoking during pregnancy	11 (3.4%)	28 (4.1%)	20 (4.9%)	1 (3.4%)	6 (3.6%)	5 (4.5%)
Passive smoking at home during childhood	188 (58.0%)	345 (50.7%)	223 (54.4%)	9 (31.0%)	86 (51.2%)	53 (48.2%)
Asthma before school age	17 (5.2%)	13 (1.9%)	22 (5.4%)	0 (0%)	8 (4.8%)	2 (1.8%)
Severe airway or lung infection before school age	86 (26.6%)	198 (28.4%)	124 (30.3%)	5 (17.2%)	42 (25.0%)	30 (27.3%)

Data are presented as No. (%) unless otherwise indicated. ICS = inhaled corticosteroid; mMRC = modified Medical Research Council; NLF = normal lung function; OAD = obstructive airway disease; PRISm = preserved ratio impaired spirometry.

a FEV_1_ to VC ratio ≥ 0.70 and FEV_1_ ≥ 80%.

b FEV_1_ to VC < 0.70.

c FEV_1_ to VC ≥ 0.70 and FEV_1_ < 80%.

d Chronic bronchitis.

### FEV_1_ Bronchodilator Response and Decline by Spirometric Pattern at First Examination

Bronchodilation testing was carried out only in individuals with decreased FEV_1_ or FEV_1_ to FVC ratio of < 0.7; hence, FEV_1_ reversibility could not be calculated for most of those with NLF. Among cases, 328 with obstruction and 143 with PRISm underwent bronchodilation testing at first examination, and the mean increase in FEV_1_ after bronchodilation was 189 mL in the group with obstruction vs 109 mL in the group with PRISm (*P* < .001). The proportion with at least 200 mL and 12% increase in FEV_1_ was 24% in the group with obstruction vs 11% in the group with PRISm (*P* = .002). Bronchodilation reclassified n = 51 (15%) and n = 50 (15%) among the 328 cases with obstruction as having NLF and PRISm, respectively, and n = 43 (30%) and n = 4 (3%) of the 143 cases with PRISm as having NLF and obstruction, respectively.

The average annual FEV_1_ decline from first examination until inclusion was greater in cases vs control participants, 42 (31) mL vs 31 (27) mL, respectively (*P* < .001). Among cases, the annual decline in FEV_1_ before bronchodilation during follow-up was 37 (35) mL in the group with obstruction and 40 (27) mL in the group with PRISm. When comparing decline in FEV_1_ during follow-up between cases and control participants with NLF, obstruction, or PRISm at first examination, it differed significantly in all comparisons (*P* < .001) (Table 2).

### PRISm and Smoking Habits at First Examination as Independent Risk Factors When Comparing Cases With Control Participants

**Figure 2:**
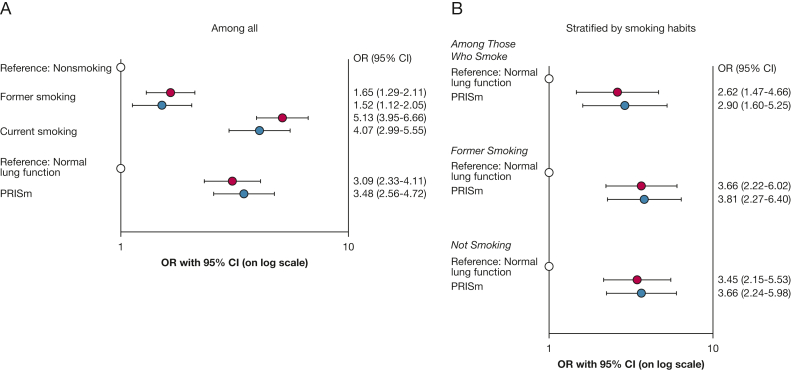
A, B, Graphs showing ORs with 95% CIs from logistic regression models comparing cases with control participants, unadjusted (red) and adjusted (blue), for smoking habits (former or current smoking, using nonsmoking as reference [white]) and spirometric patterns (PRISm, using normal spirometry findings as reference [white]) at first examination: among all individuals (A) and stratified by smoking habits (B). Outcome: airway obstruction (ie, being a case in the OLIN COPD Study, control participants as reference). Unadjusted: adjusted for cohort only. Adjusted: with spirometric patterns, age, sex, smoking habits, BMI, and cohort at first examination included as covariates in the model. PRISm = preserved ratio impaired spirometry.

Both former smoking (adjusted OR [aOR], 1.52) and current smoking (aOR, 4.07) at first examination were associated with classification as a case at recruitment of the COPD study (using nonsmoking as reference), as were PRISm, yielding an aOR of 3.48 (using NLF as reference). Stratification by smoking habits yielded similar aORs for PRISm among the current smoking group (aOR, 2.90), former smoking group (aOR, 3.81), and nonsmoking group (aOR, 3.66), respectively (Fig 2).

Results stratified by sex are presented in e-Table 1 and yielded similar findings in males and females. Also, in the sensitivity analyses including spirometry findings after bronchodilation to define cases, a similar pattern as described remained (e-Table 2). Further, adjustment also for inhaled corticosteroid use at first examination did not change the main findings (results not shown), and none of the interaction terms between PRISm and other covariates yielded significance (e-Table 3).

### Additional Analyses Including RSP

**Figure 3:**
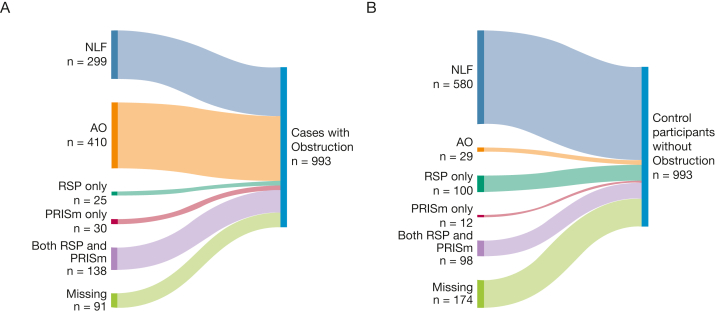
A, B, Illustrations showing distribution by spirometric pattern among cases (A) and control participants (B): NLF, AO, RSP only, PRISm only, and both RSP and PRISm or missing at first examination. AO = airway obstruction; NLF = normal lung function; PRISm = preserved ratio impaired spirometry; RSP = restrictive spirometric pattern.

**Figure 4:**
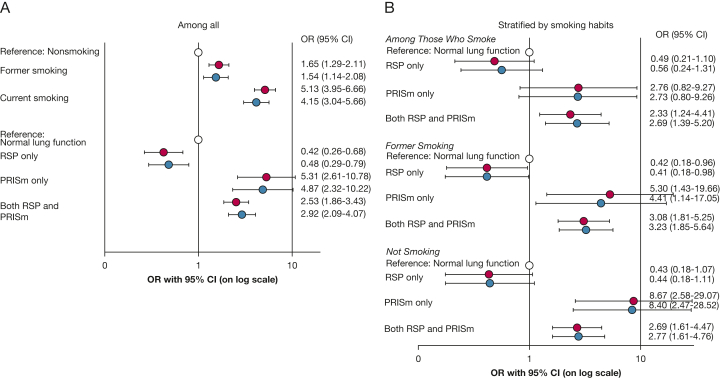
A, B, Graphs showing ORs with 95% CIs from logistic regression models comparing cases with control participants, unadjusted (red) and adjusted (blue), for smoking habits (former or current smoking, using nonsmoking as reference [white]) and spirometric patterns (RSP only, PRISm only, or both RSP and PRISm, using normal spirometry findings as reference [white]) at first examination among all participants (A) and stratified by smoking habits (B). Outcome: airway obstruction (ie, being a case in the OLIN COPD Study, control participants as reference). Unadjusted: adjusted for cohort only. Adjusted: with spirometric patterns, age, sex, smoking habits, BMI, and cohort at first examination as covariates in the model. In stratified analyses, smoking habits were not included as covariates. PRISm = preserved ratio impaired spirometry; RSP = restrictive spirometric pattern.

Substantial overlap was found between RSP and PRISm in both cases and control participants but was most pronounced among cases (Fig 3A, 3B). Clinical characteristics by spirometric groups including RSP are presented in e-Tables 4A and 4B among cases and control participants, respectively. Having PRISm only (without concomitant RSP) yielded an aOR of 4.87, and having PRISm plus RSP yielded an aOR of 2.92 for classification as a case at recruitment of the COPD study (Fig 4). By contrast, the aOR for RSP only (without concomitant PRISm) was 0.48. Stratification by smoking habits yielded similar aORs for all spirometric patterns except for PRISm only, with aORs of 2.73 in the current smoking group, 4.41 in the former smoking group, and 8.40 in the nonsmoking group (Fig 4). The pattern was similar in analyses stratified by sex (e-Table 5) and also in the sensitivity analyses, where the outcome case was defined to include spirometry after bronchodilation (e-Table 6).

## Discussion

The longitudinal follow-ups provide an excellent opportunity to evaluate so-called pre-COPD in the current study by linking previous clinical data, on average a decade earlier, to the cases and control participants of the OLIN COPD Study. As expected, AO and both former and current smoking already at first examination were associated strongly with becoming an included case. Importantly, PRISm indeed had a pre-COPD status, because PRISm clearly associated with transition to AO, an association of similar strength as that of current smoking status.

PRISm previously was shown to be associated with both smoking and development of COPD,^19^ but importantly, our results significantly support an association between PRISm and development of AO not only in those who smoke, but also in those who do not smoke. Only a few studies have had sufficient power to enable stratification by smoking habits, and our findings add important knowledge to the concept of pre-COPD. The results clearly show that smoking is not the only explanation for an association between PRISm and development of COPD; other factors also are likely at play. Impaired lifetime FEV_1_ trajectories have been associated with early life events such as RSV infections, childhood asthma, low birth weight, and being small for gestational age, as well as with environmental and genetic or epigenetic factors.^20^, ^21^, ^22^ However, we could not confirm any differences in the limited data on early life events, childhood asthma, or bronchodilation response between those with PRISm and those with obstruction at first examination. The *Lancet* Commission article on COPD^23^ is of importance in this context, highlighting also other phenotypes besides the smoking-induced COPD and the importance of identifying early pathologic changes that may precede irreversible changes and AO.

At the first examination of future cases with AO, individuals presenting with PRISm and AO had a similar burden of respiratory symptoms and medication use along with similar rate of FEV_1_ decline during follow-up, and these similarities may contribute to misclassification of COPD.^24^ Also, those with PRISm in the COPDGene Study showed a higher exacerbation frequency and higher burden of comorbidities than those without airflow obstruction at baseline,^25^ that is, the same profile as in COPD. In clinical practice, PRISm may indeed be treated or diagnosed as COPD, or both.^26^^,^^27^ Importantly, in our retrospective case-control study, we found that PRISm that transitioned into AO presented with more chronic bronchitis, dyspnea, and wheeze and a more rapid decline in FEV_1_ than PRISm that did not transition into AO during follow-up. A Chinese population-based study also indicated that the effect of how well PRISm predicts COPD may be dependent of chronic bronchitis and small airways dysfunction.^28^ An important research question is whether subtypes of PRISm representing early pathologic changes that portend COPD exist, and whether treatment can prevent progression to COPD.

PRISm is considered an unstable spirometric pattern. Transition into and out of PRISm is common,^29^ and a few prospective studies have evaluated risk factors for and transition into COPD. In a 5-year follow-up of the COPDGene Study (including only those who have ever smoked) around 1 in 4 of those with PRISm demonstrated AO.^30^ Transition into COPD was associated with a higher burden of smoking, but also a higher loss of FEV_1_. After 10 years of follow-up, 65% of those who transitioned out of PRISm demonstrated obstruction.^29^ In a population-based Korean study, 13.6% of those with PRISm demonstrated COPD after 2 years and after 4 years; in total around 18% had transitioned to COPD.^31^ In the population-based Proyecto LatinoAmericano de Investigación en Obstrucción Pulmonar study, 18% of those with PRISm transitioned into COPD after 5 to 9 years, and in addition to age, current smoking was associated with this change.^32^ A Danish study including 1,160 randomly selected individuals 20 to 40 years of age at baseline followed up for > 3 decades confirmed an association of PRISm with smoking and an increased risk of both COPD admissions and all-cause mortality.^19^ To summarize, transition from PRISm to COPD is often associated with smoking; however, previous analyses stratified for smoking habits comparable with the current study are lacking.

PRISm is associated with increased mortality.^32^^,^^33^ Furthermore, RSP defined by low FVC is associated with worse prognosis assessed as mortality, an association that is similar in COPD.^34^^,^^35^ Also, causes of death in those with PRISm (in the COPDGene Study^33^) and in those with RSP (the OLIN COPD study^35^) seem to have a similar pattern, although the population selection differs between studies. The terms RSP and PRISm are sometimes used interchangeably^7^^,^^19^ or are simply covered by the term *pulmonary restriction*, and the reported prevalences are quite similar: RSP in the range of 7% to 11%^36^ and PRISm in the range of 7% to 13%,^37^ with a stable prevalence over time.^34^^,^^38^ Smoking has been recognized as a risk factor for both conditions.^7^^,^^19^^,^^39^, ^40^, ^41^ A recent publication based on cross-sectional data has reported a substantial overlap between RSP and PRISm in a Swedish population sample 55 to 65 years of age,^10^ and the current study also showed a large overlap, especially among cases with future AO. Interestingly, according to our results, the association with future AO seems to be driven by PRISm, whereas RSP only was less common among future cases than control participants. This pattern was stable also when stratifying for smoking habits and sex, and it persisted in the sensitivity analyses based on spirometry values after bronchodilation. Thus, RSP and PRISm seem to represent different clinical profiles, supported also by a recently published prospective study (including men only) assessing prognosis by mortality in which RSP, but not PRISm, showed an increased risk for all-cause mortality, although those with RSP plus PRISm showed the worst prognosis.^42^ Also the cause-specific mortality patterns differed between spirometric groups in that study.^42^ Evidently a need exists to disentangle the clinical implication of RSP, PRISm, or both in relationship to pre-COPD and transition into AO, as well as in a wider context. Future studies are needed to evaluate possible underlying biological mechanisms reflected by different biomarker profiles among those with RSP and PRISm, further associated with development toward COPD and other prognostic outcomes.

Our study extends the current literature through its design, with about 10 years of retrospective population-based data on spirometry, smoking habits, respiratory symptom profiles, and early life events for around 900 adults with AO. This large COPD cohort provided a solid basis for analysis enabling stratification by smoking habits. The age-matched and sex-matched control group enabled comparisons with individuals not demonstrating AO unbiased by these characteristics. Further, lung function testing was carried out with a uniform methodology following contemporary guidelines and using the same set of spirometers throughout the study period. The additional analyses including mutually exclusive spirometric restrictive groups—PRISm only, RSP only, and PRISm plus RSP—did contribute hypothesis-generating results for future studies on the importance of spirometric patterns in relationship to the concept of pre-COPD and transition to AO.

However, limitations must also be considered. First, our main analyses were based on spirometry results before bronchodilation. Yet, in the sensitivity analyses including spirometry after bronchodilation, the major findings remained, that is, the association of PRISm with the development of AO. Moreover, some transition between spirometric groups was observed. Second, lower limit of normal and *z* score were not applied so as not to violate the original study design of the OLIN COPD Study using a fixed ratio to define AO. Third, PRISm has been described as being common in diabetes,^43^ but we lack data on comorbidities at the first examination beyond information on ischemic heart disease. Fourth, the sample size may not be adequate to detect meaningful associations, and small sample size may introduce type II error and instable models. However, the stratification by smoking habits showed similar patterns of associations for PRISm in those who did not smoke, those who formerly smoked, and those who currently smoke as in the entire sample, implying model stability in subgroups. Because of the hypothesis-generating design of this study, we did not perform adjustment for multiple testing. Thus, the results should be interpreted keeping also the risk of type I errors in mind. Finally, we lack information on risk factors for COPD other than smoking, for example, such as occupational exposure and socioeconomic status, as well as on data necessary to enable endotyping and genetic analyses. Furthermore, the study population is primarily White which should be considered in terms of generalizability of the results.

## Interpretation

In this retrospective population-based study, PRISm was associated with transition into AO when evaluated with on average 10 years of follow-up. The association was independent of smoking habits but, not shown previously, of similar strength among those who did not smoke, those who formerly smoked, and those who currently smoke. Our findings warrant further studies to explore potential underlying disease mechanisms that may be associated with spirometric patterns and the development of COPD. Further knowledge on PRISm may reveal potential preventive measures against COPD, beyond the mainstay of smoking cessation.

## Funding/Support

The study was supported by grants from the Swedish Heart and Lung Foundation, a regional agreement between Umea University and Region Vasterbotten, the Swedish Respiratory Society, VISARE NORR Fund Northern County Councils Regional Federation, and the 10.13039/501100009772Norrbotten County Council.

## Financial/Nonfinancial Disclosures

The authors have reported to *CHEST* the following: H. B. reports personal fees from Chiesi, outside the submitted work. C. S. reports personal fees from GlaxoSmithKline, AstraZeneca, Chiesi, and TEVA and fees for advisory board work from AstraZeneca and GlaxoSmithKline, all outside the submitted work. A. L. reports personal fees from AstraZeneca and fees for advisory board work from AstraZeneca, 10.13039/100004330GlaxoSmithKline, Boehringer Ingelheim, and Novartis, all outside the submitted work. None declared (T. M., L. H., E. R.).

## Supplementary Data

e-Online Data

e-Online Data

e-Online Data

e-Online Data

## References

[1] Adeloye D., Song P., Zhu Y. (2022). Global, regional, and national prevalence of, and risk factors for, chronic obstructive pulmonary disease (COPD) in 2019: a systematic review and modelling analysis. Lancet Respir Med.

[2] Boers E., Barrett M., Su J.G. (2023). Burden of chronic obstructive pulmonary disease through 2050. JAMA Netw Open.

[3] Siddharthan T., Grigsby M.R., Goodman D. (2018). Association between household air pollution exposure and chronic obstructive pulmonary disease outcomes in 13 low- and middle-income country settings. Am J Respir Crit Care Med.

[4] Lamprecht B., Soriano J.B., Studnicka M. (2015). Determinants of underdiagnosis of COPD in national and international surveys. Chest.

[5] Axelsson M., Backman H., Nwaru B.I. (2023). Underdiagnosis and misclassification of COPD in Sweden: a Nordic Epilung study. Respir Med.

[6] Global Initiative for Chronic Obstructive Lung Disease, About GOLD. Global Initiative for Chronic Obstructive Lung Disease website. http://www.goldcopd.com.

[7] Guerra S., Sherrill D.L., Venker C., Ceccato C.M., Halonen M., Martinez F.D. (2010). Morbidity and mortality associated with the restrictive spirometric pattern: a longitudinal study. Thorax.

[8] Wijnant S.R.A., De Roos E., Kavousi M. (2020). Trajectory and mortality of preserved ratio impaired spirometry: the Rotterdam Study. Eur Respir J.

[9] Wan E.S., Balte P., Schwartz J.E. (2021). Association between preserved ratio impaired spirometry and clinical outcomes in US adults. JAMA.

[10] Torén K., Blomberg A., Schiöler L. (2024). Restrictive spirometric pattern and preserved ratio impaired spirometry in a population 50-64 years. Ann Am Thorac Soc.

[11] Lindberg A., Lundbäck B. (2008). The Obstructive Lung Disease in Northern Sweden Chronic Obstructive Pulmonary Disease Study: design, the first year participation and mortality. Clin Respir J.

[12] von Elm E., Altman D.G., Egger M. (2008). The Strengthening the Reporting of Observational Studies in Epidemiology (STROBE) statement: guidelines for reporting observational studies. J Clin Epidemiol.

[13] (1979). ATS statement—Snowbird workshop on standardization of spirometry. Am Respir Dis.

[14] American Thoracic Society (1995). Standardization of spirometry, 1994 update. Am J Respir Crit Care Med.

[15] Backman H., Lindberg A., Odén A. (2015). Reference values for spirometry—report from the Obstructive Lung Disease in Northern Sweden studies. Eur Clin Respir J.

[16] Lundbäck B., Stjernberg N., Nyström L. (1994). Epidemiology of respiratory symptoms, lung function and important determinants. Report from the Northern Sweden Obstructive Lung Disease Project. Tuber Lung Dis.

[17] Lâm H.T., Ekerljung L., Tu'ò'ng N.V., Rönmark E., Larsson K., Lundbäck B. (2014). Prevalence of COPD by disease severity in men and women in northern Vietnam. COPD.

[18] Jalasto J., Lassmann-Klee P., Schyllert C. (2022). Occupation, socioeconomic status and chronic obstructive respiratory diseases—the EpiLung study in Finland, Estonia and Sweden. Respir Med.

[19] Marott J.L., Ingebrigtsen T.S., Çolak Y., Vestbo J., Lange P. (2021). Trajectory of preserved ratio impaired spirometry: natural history and long-term prognosis. Am J Respir Crit Care Med.

[20] Svanes C., Sunyer J., Plana E. (2010). Early life origins of chronic obstructive pulmonary disease. Thorax.

[21] Bui D.S., Lodge C.J., Burgess J.A. (2018). Childhood predictors of lung function trajectories and future COPD risk: a prospective cohort study from the first to the sixth decade of life. Lancet Respir Med.

[22] Melén E., Faner R., Allinson J.P. (2024). Lung-function trajectories: relevance and implementation in clinical practice. Lancet.

[23] Stolz D., Mkorombindo T., Schumann D.M. (2022). Towards the elimination of chronic obstructive pulmonary disease: a Lancet Commission. Lancet.

[24] Sator L., Horner A., Studnicka M. (2019). Overdiagnosis of COPD in subjects with unobstructed spirometry: a BOLD analysis. Chest.

[25] Parekh T.M., Bhatia S., Cherrington A. (2020). Factors influencing decline in quality of life in smokers without airflow obstruction: the COPDGene Study. Respir Med.

[26] Agustí A., Hughes R., Rapsomaki E. (2024). The many faces of COPD in real life: a longitudinal analysis of the NOVELTY cohort. ERJ Open Res.

[27] Vanfleteren L.E.G.W., Lindberg A., Zhou C., Nyberg F., Stridsman C. (2023). Exacerbation risk and mortality in Global Initiative for Chronic Obstructive Lung Disease group A and B patients with and without exacerbation history. Am J Respir Crit Care Med.

[28] Fan J., Fang L., Cong S. (2023). Potential pre-COPD indicators in association with COPD development and COPD prediction models in Chinese: a prospective cohort study. Lancet Reg Health West Pac.

[29] Wan E.S., Hokanson J.E., Regan E.A. (2022). Significant spirometric transitions and preserved ratio impaired spirometry among ever smokers. Chest.

[30] Wan E.S., Fortis S., Regan E.A. (2018). Longitudinal phenotypes and mortality in preserved ratio impaired spirometry in the COPDGene Study. Am J Respir Crit Care Med.

[31] Jo Y.S., Rhee C.K., Kim S.H., Lee H., Choi J.Y. (2024). Spirometric transition of at risk individuals and risks for progression to chronic obstructive pulmonary disease in general population. Arch Bronconeumol.

[32] Perez-Padilla R., Montes de Oca M., Thirion-Romero I. (2023). Trajectories of spirometric patterns, obstructive and PRISm, in a population-based cohort in Latin America. Int J Chron Obstruct Pulmon Dis.

[33] Labaki W.W., Gu T., Murray S. (2023). Causes of and clinical features associated with death in tobacco cigarette users by lung function impairment. Am J Respir Crit Care Med.

[34] Cadham C.J., Oh H., Han M.K. (2024). The prevalence and mortality risks of PRISm and COPD in the United States from NHANES 2007-2012. Respir Res.

[35] Backman H., Sawalha S., Nilsson U. (2024). All-cause and cause-specific mortality by spirometric pattern and sex—a population-based cohort study. Ther Adv Respir Dis.

[36] Backman H., Eriksson B., Hedman L. (2016). Restrictive spirometric pattern in the general adult population: methods of defining the condition and consequences on prevalence. Respir Med.

[37] Huang J., Li W., Sun Y. (2024). Preserved ratio impaired spirometry associations, and differentiation from chronic obstructive pulmonary disease. Int J Chron Obstruct Pulmon Dis.

[38] Choi H., Oak C.H., Jung M.H., Jang T.W., Nam S.J., Yoon T. (2024). Trend of prevalence and characteristics of preserved ratio impaired spirometry (PRISm): nationwide population-based survey between 2010 and 2019. PLoS One.

[39] Lederer D.J., Enright P.L., Kawut S.M. (2009). Cigarette smoking is associated with subclinical parenchymal lung disease: the Multi-Ethnic Study of Atherosclerosis (MESA)-lung study. Am J Respir Crit Care Med.

[40] Wan E.S., Castaldi P.J., Cho M.H. (2014). Epidemiology, genetics, and subtyping of preserved ratio impaired spirometry (PRISm) in COPDGene. Respir Res.

[41] Schiffers C., Ofenheimer A., Breyer M.K. (2023 Apr-May). Prevalence of restrictive lung function in children and adults in the general population. Respir Med.

[42] Cestelli L., Johannessen A., Gulsvik A., Stavem K., Nielsen R. (2025). Risk factors, morbidity and mortality in association with Preserved Ratio Impaired Spirometry (PRISm) and Restrictive Spirometric Pattern (RSP). Chest.

[43] Li G., Jankowich M.D., Wu L., Lu Y., Shao L., Lu X., Fan Y., Pan C.W., Wu Y., Ke C. (2023). Preserved ratio impaired spirometry and risks of macrovascular, microvascular complications and mortality among individuals with type 2 diabetes. Chest.

