# Supplementary material for: Importance of Preserved Ratio Impaired Spirometry as a Risk Factor for Development of COPD, Also in Those Who Do Not Smoke
Source: Chest. 2025 Mar 8;168(1):83–94. doi: 10.1016/j.chest.2025.02.025 (PMC12264347; doi:10.1016/j.chest.2025.02.025)
Supplement: e-Online Data [file mmc2.pdf]

Questionnaire survey	First clinical examination	Recruitment to the OLIN COPD study	
Four (I-IV) population-based cohorts [examination year]. N=survey responders.	Clinical examinations of population-based cohorts I-III [examination year]. N=individuals with spirometry data*	Clinical examinations of four (I-IV) population-based cohorts [examination year].	
Cohort I n=508 [1985 and 1996]<br>Cohort II n=291 [1992]<br>Cohort III n=121 [1992]<br>Cohort IV n=73 [1996]<br>**Total n=993**	Cohort I n=490 [1986 or 1996]<br>Cohort II n=291 [1996]<br>Cohort III n=121 [1994-1995]<br>*Cohort IV [no previous clinical ex]*<br>**Total n=902***	Cohort I n=508 [2002-2004]<br>Cohort II n=291 [2002-2004]<br>Cohort III n=121 [2002-2004]<br>Cohort IV n=73 [1997-2001]<br>**Total n=993**	**N=993 pre-bd obstructive cases (FEV1/VC<0.7) recruited**
Cohort I n=510 [1985 and 1996]<br>Cohort II n=101 [1992]<br>Cohort III n=234 [1992]<br>Cohort IV n=148 [1996]<br>**Total n=993**	Cohort I n=485 [1986 or 1996]<br>Cohort II n=101 [1996]<br>Cohort III n=233 [1994-1995]<br>*Cohort IV [no previous clinical ex]*<br>**Total n=819***	Cohort I n=510 [2002-2004]<br>Cohort II n=101 [2002-2004]<br>Cohort III n=234 [2002-2004]<br>Cohort IV n=148 [1997-2001]<br>**Total n=993**	**N=993 pre-bd non-obstructive age- and sex-matched controls (FEV1/VC ≥ 0.7) recruited**

Individuals with spirometry data at a first clinical examination prior to recruitment to the OLIN COPD Study

N=902 cases* **N=819 controls***

* Complete data on spirometry, age, sex and height.
