# Supplementary material for: Importance of Preserved Ratio Impaired Spirometry as a Risk Factor for Development of COPD, Also in Those Who Do Not Smoke
Source: Chest. 2025 Mar 8;168(1):83–94. doi: 10.1016/j.chest.2025.02.025 (PMC12264347; doi:10.1016/j.chest.2025.02.025)
Supplement: e-Online Data [file mmc3.docx]

e-Table 1. Association between spirometric patterns at first examination (airway obstruction and PRISm, using normal lung function as reference) and being a case (i.e. having airway obstruction) at inclusion to the OLIN COPD study, expressed as Odds Ratios (OR) with 95% confidence intervals (CI) from logistic regression models, stratified by sex.

|  | **Unadjusted^1^** | | | **Adjusted^2^** | | |
| --- | --- | --- | --- | --- | --- | --- |
| Pre-bd spirometric pattern at first examination |  |  | |  |  | |
|  | **OR** | **95% CI** | | **OR** | **95% CI** | |
| *Among women* |  |  |  |  |  |  |
| Normal lung function | 1 |  |  | 1 |  |  |
| Airway obstruction | 27.67 | 14.73 | 51.98 | 24.77 | 13.02 | 47.12 |
| PRISm | 3.11 | 2.09 | 4.63 | 3.30 | 2.18 | 5.01 |
|  |  |  |  |  |  |  |
| *Among men* |  |  |  |  |  |  |
| Normal lung function | 1 |  |  | 1 |  |  |
| Airway obstruction | 30.58 | 18.02 | 51.91 | 35.61 | 20.31 | 62.44 |
| PRISm | 2.96 | 1.96 | 4.46 | 3.80 | 2.41 | 5.99 |

^1^adjusted for cohort, ^2^adjusted for age, smoking habits, BMI and cohort

Normal Lung Function (NLF; FEV_1_/VC>0.70 & FEV_1_>80% of predicted)

Pre-bd=Pre-bronchodilation

e-Table 2. Association between smoking habits and spirometric patterns at first examination (airway obstruction and PRISm, using normal lung function as reference) and being a case (i.e. having post-bd airway obstruction) at inclusion to the OLIN COPD study, expressed as Odds Ratios (OR) with 95% confidence intervals (CI) from logistic regression models.

|  | **Unadjusted^1^** | | | **Adjusted^2^** | | |
| --- | --- | --- | --- | --- | --- | --- |
| Pre-bd spirometric pattern and smoking habits at first examination | | | |  |  | |
|  | **OR** | **95% CI** | | **OR** | **95% CI** | |
| *Smoking habits* |  |  |  |  |  |  |
| Non-smoker | 1 |  |  | 1 |  |  |
| Former smoker | 1.68 | 1.27 | 2.21 | 1.45 | 1.02 | 2.06 |
| Current smoker | 6.20 | 4.67 | 8.23 | 4.88 | 3.44 | 6.91 |
|  |  |  |  |  |  |  |
| *Spirometric patterns* |  |  |  |  |  |  |
| Normal lung function | 1 |  |  | 1 |  |  |
| Airway obstruction | 37.72 | 24.91 | 57.12 | 36.58 | 23.68 | 56.52 |
| PRISm | 3.34 | 2.44 | 4.56 | 3.71 | 2.64 | 5.22 |

^1^adjusted for cohort, ^2^adjusted for spirometric patterns, age, sex, smoking habits, BMI and cohort

Normal Lung Function (NLF; FEV_1_/VC>0.70 & FEV_1_>80% of predicted)

Pre-bd=Pre-bronchodilation

e-Table 3. Odds Ratios (OR) with 95% confidence intervals (CI) from logistic regression models for interaction terms between pre-bronchodilatory PRISm and other model covariates.

| **Covariate** | **OR** | **95% CI** | |
| --- | --- | --- | --- |
| Age | 1.00 | 0.97 | 1.03 |
| Sex | 1.00 | 0.55 | 1.83 |
| Body Mass Index | 1.02 | 0.95 | 1.09 |
| ICS use | 0.97 | 0.25 | 3.72 |
| Former smoker | 1.10 | 0.54 | 2.22 |
| Current smoker | 0.73 | 0.35 | 1.55 |

ICS=Inhaled corticosteroid

e-Table 4A. Clinical characteristics and comorbidities at the first examination by pre-bronchodilatory spirometric patterns (NLF, Airway Obstruction (AO), RSP only, PRISm only and RSP & PRISm) among cases, n=902.

| **Variables** | **NLF** | **AO** | **RSP only** | **PRISm only** | **RSP & PRISm** |
| --- | --- | --- | --- | --- | --- |
|  | n= 299 | n= 410 | n=25 | n=30 | n=138 |
| **Demographics** |  |  |  |  |  |
| Age, years, mean (SD) | 52.1 (11.5) | 78.7 (11.4) | 58.6 (9.1) | 66.4 (10.6) | 57.6 (9.6) |
| Sex, women | 132 (44.1) | 168 (41.0) | 13 (52.0) | 16 (53.3) | 78 (56.5) |
| Body mass index, kg/m^2^, mean (SD) | 25.3 (3.6) | 25.1 (3.4) | 26.1 (3.9) | 24.8 (3.6) | 26.6 (4.7) |
| **Smoking habits** |  |  |  |  |  |
| Non-smoker | 74 (24.7) | 81 (19.8) | 6 (24.0) | 10 (33.3) | 37 (26.8) |
| Former smoker | 85 (28.4) | 137 (33.4) | 8 (32.0) | 7 (23.3) | 50 (36.2) |
| Current smoker | 140 (46.8) | 192 (46.8) | 11 (44.0) | 13 (43.3) | 51 (37.0) |
| **Respiratory symptoms, diagnosis and medication** | |  |  |  |  |
| ^1^Productive cough 3 months/year at least 2 years | 110 (36.8) | 186 (45.4) | 8 (32.0) | 9 (30.0) | 74 (53.6) |
| Dyspnea (mMRC ≥ 2) | 47 (15.7) | 103 (25.1) | 5 (20.0) | 4 (13.3) | 39 (28.3) |
| Wheeze last 12 months | 179 (59.9) | 295 (72.0) | 14 (56.0) | 20 (66.7) | 99 (71.7) |
| Any Respiratory symptoms | 214 (71.6) | 335 (81.7) | 19 (76.0) | 22 (73.3) | 114 (82.6) |
| Family history of asthma | 76 (25.4) | 131 (32.0) | 7 (28.0) | 9 (30.0) | 35 (25.4) |
| ^2^PD asthma ever | 42 (14.0) | 115 (28.0) | 4 (16.0) | 8 (26.7) | 33 (23.9) |
| PD chronic bronchitis or emphysema | 43 (14.4) | 76 (18.5) | 4 (16.0) | 3 (10.0) | 27 (19.6) |
| Airway medication for ^3^OAD last 12 months | 46 (15.4) | 130 (31.7) | 4 (16.0) | 7 (23.3) | 42 (30.4) |
| **Comorbidity** |  |  |  |  |  |
| Ischemic heart disease | 35 (11.7) | 53 (12.9) | 6 (24.0) | 0 (0) | 22 (15.9) |
| **Early life events** |  |  |  |  |  |
| Maternal smoking during pregnancy | 10 (3.3) | 20 (4.9) | 1 (4.0) | 1 (3.4) | 5 (3.6) |
| Parental smoking | 176 (59.1) | 223 (54.5) | 12 (48.0) | 19 (63.3) | 67 (48.9) |
| Asthma before school age | 15 (5.0) | 22 (5.4) | 2 (8.0) | 5 (16.7) | 3 (2.2) |
| Severe airway/lung infection before school age | 80 (26.8) | 124 (30.3) | 6 (24.0) | 7 (23.3) | 25 (25.4) |

^1^Chronic bronchitis, ^2^Physician Diagnosis, ^3^Obstructive Airway Disease, ^4^Preserved Ratio Impaired Spirometry

Normal Lung Function (NLF; FEV_1_/VC>0.70 & FEV_1_>80% of predicted & FVC>80% of predicted)

e-Table 4B. Clinical characteristics and comorbidities at the first examination by pre-bronchodilatory spirometric patterns (NLF, Airway Obstruction (AO), RSP only, PRISm only and RSP & PRISm) among controls (n=819).

| **Variables** | **NLF** | **AO** | **RSP only** | **PRISm only** | **RSP & PRISm** |
| --- | --- | --- | --- | --- | --- |
|  | n=580 | n=29 | n=100 | n=12 | n=98 |
| **Demographics** |  |  |  |  |  |
| Age, years, mean (SD) | 54.6 (11.5) | 62.3 (11.2) | 61.0 (9.3) | 53.2 (9.9) | 59.8 (8.7) |
| Sex, women | 246 (42.4) | 12 (41.4) | 51 (51.0) | 5 (41.7) | 54 (55.1) |
| Body mass index, kg/m^2^, mean (SD) | 25.6 (3.2) | 27.1 (3.8) | 26.8 (4.4) | 53.2 (9.9) | 26.4 (4.5) |
| **Smoking habits** |  |  |  |  |  |
| Non-smoker | 276 (47.6) | 11 (37.9) | 45 (45.0) | 4 (33.3) | 48 (49.0) |
| Former smoker | 200 (34.5) | 12 (41.4) | 37 (37.0) | 4 (33.3) | 33 (33.7) |
| Current smoker | 104 (17.9) | 6 (20.7) | 18 (18.0) | 4 (33.3) | 17 (17.3) |
| **Respiratory symptoms, diagnosis and medication** | |  |  |  |  |
| Productive cough, 3 months/year | 111 (19.1) | 9 (31.0) | 23 (23.0) | 4 (33.3) | 33 (33.7) |
| ^1^Productive cough 3 months/year at least 2 years | 95 (16.4) | 9 (31.0) | 18 (18.0) | 4 (33.3) | 32 (32.7) |
| Dyspnea (mMRC ≥ 2) | 38 (6.6) | 3 (10.3) | 16 (16.0) | 0 (0) | 18 (18.4) |
| Wheeze last 12 months | 160 (27.6) | 12 (41.4) | 29 (29.0) | 6 (50.0) | 57 (58.2) |
| Any Respiratory symptoms | 228 (39.3) | 17 (58.6) | 48 (48.0) | 7 (58.3) | 63 (64.3) |
| Family history of asthma | 130 (22.4) | 6 (20.7) | 23 (23.0) | 3 (25.0) | 24 (24.5) |
| ^2^PD asthma ever | 37 (6.4) | 4 (13.8) | 6 (6.0) | 1 (8.3) | 14 (14.3) |
| PD chronic bronchitis or emphysema | 28 (4.8) | 0 (0) | 8 (8.0) | 0 (0) | 7 (7.1) |
| Airway medication for ^3^OAD last 12 months | 37 (6.4) | 3 (10.3) | 8 (8.0) | 1 (8.3) | 14 (14.3) |
| **Comorbidity** |  |  |  |  |  |
| Ischemic heart disease | 35 (6.0) | 5 (17.2) | 13 (13.0) | 1 (8.3) | 15 (15.3) |
| **Early life events** |  |  |  |  |  |
| Maternal smoking during pregnancy | 27 (4.7) | 1 (3.4) | 1 (1.0) | 1 (2.9) | 4 (4.1) |
| Parental smoking at home during childhood | 301 (52.2) | 9 (31.0) | 44 (44.4) | 8 (66.7) | 45 (46.4) |
| Asthma before school age | 13 (2.2) | 0 (0) | 0 (0) | 0 (0) | 2 (2.0) |
| Severe airway/lung infection before school age | 164 (28.3) | 5 (17.2) | 29 (29.0) | 3 (25.0) | 27 (27.6) |

^1^Chronic bronchitis, ^2^Physician Diagnosis, ^3^Obstructive Airway Disease

Normal Lung Function (NLF; FEV_1_/VC>0.70 & FEV_1_>80% of predicted & FVC>80% of predicted)

e-Table 5. Association between spirometric patterns at first examination (airway obstruction, RSP only, PRISm only and RSP & PRISm, using normal lung function as reference) and being a case (i.e. having airway obstruction) at inclusion to the OLIN COPD study, expressed as Odds Ratios (OR) with 95% confidence intervals (CI) from logistic regression models, stratified by sex.

|  | Unadjusted^1^ | | | Adjusted^2^ | | |
| --- | --- | --- | --- | --- | --- | --- |
| Pre-bd spirometric pattern at first examination |  |  | |  |  | |
|  | OR | 95% CI | | OR | 95% CI | |
| *Among women* |  |  |  |  |  |  |
| Normal lung function | 1 |  |  | 1 |  |  |
| Airway obstruction | 24.65 | 13.05 | 46.58 | 22,26 | 11.63 | 42.60 |
| RSP only | 0.37 | 0.19 | 0.73 | 0.45 | 0.23 | 0.90 |
| PRISm only | 6.35 | 2.16 | 18.71 | 6.19 | 2.00 | 19.14 |
| RSP & PRISm | 2.44 | 1.59 | 3.73 | 2.66 | 1.70 | 4.15 |
|  |  |  |  |  |  |  |
| *Among men* |  |  |  |  |  |  |
| Normal lung function | 1 |  |  | 1 |  |  |
| Airway obstruction | 28.40 | 16.68 | 48.37 | 32.47 | 18.44 | 57.19 |
| RSP only | 0.45 | 0.23 | 0.89 | 0.49 | 0.24 | 1.00 |
| PRISm only | 4.47 | 1.73 | 11.55 | 4.21 | 1.53 | 11.61 |
| RSP & PRISm | 2.49 | 1.59 | 3.89 | 3.33 | 2.02 | 5.50 |

^1^adjusted for cohort, ^2^adjusted for age, smoking habits, BMI and cohort

Normal Lung Function (NLF; FEV_1_/VC>0.70 & FEV_1_>80% of predicted & FVC>80% of predicted)

e-Table 6. Association between smoking habits and spirometric patterns at first examination (airway obstruction, RSP only, PRISm only and RSP & PRISm, using normal lung function as reference) and being a case defined as having post-bronchodilatory airway obstruction at inclusion to the OLIN COPD study, expressed as Odds Ratios (OR) with 95% confidence intervals (CI) from logistic regression models.

|  | Unadjusted^1^ | | | Adjusted^2^ | | |
| --- | --- | --- | --- | --- | --- | --- |
| Pre-bd spirometric pattern and smoking habits at first examination | | | |  |  | |
|  | OR | 95% CI | | OR | 95% CI | |
| *Smoking habits* |  |  |  |  |  |  |
| Non-smoker | 1 |  |  | 1 |  |  |
| Former smoker | 1.68 | 1.27 | 2.21 | 1.47 | 1.03 | 2.08 |
| Current smoker | 6.20 | 4.67 | 8.23 | 4.95 | 3.48 | 7.03 |
|  |  |  |  |  |  |  |
| *Spirometric pattern* |  |  |  |  |  |  |
| Normal lung function | 1 |  |  | 1 |  |  |
| Airway obstruction | 34.37 | 22.62 | 52.22 | 33.06 | 21.31 | 51.27 |
| RSP only | 0.41 | 0.23 | 0.73 | 0.46 | 0.25 | 0.83 |
| PRISm only | 5.57 | 2.59 | 11.95 | 4.91 | 2.19 | 10.99 |
| RSP & PRISm | 2.75 | 1.97 | 3.85 | 3.15 | 2.18 | 4.56 |

^1^adjusted for cohort, ^2^adjusted for spirometric patterns, age, sex, smoking habits, BMI and cohort.

Pre-bd=Pre-bronchodilation
