# Supplementary material for: Importance of Preserved Ratio Impaired Spirometry as a Risk Factor for Development of COPD, Also in Those Who Do Not Smoke
Source: Chest. 2025 Mar 8;168(1):83–94. doi: 10.1016/j.chest.2025.02.025 (PMC12264347; doi:10.1016/j.chest.2025.02.025)
Supplement: e-Online Data [file mmc4.docx]

**Caption for e-Figure 1**

e-Figure 1 provides an overview of the calendar years and number of participants for the surveys and clinical examinations of participants in the OLIN COPD Study. In 2002-04, previously examined individuals from the OLIN adult cohorts I-IV were invited to re-examinations, after which all individuals with airway obstruction (FEV1/VC<0.70, n=993 cases) were identified together with age- and sex matched controls without airway obstruction (FEV1/VC>0.70, n=993 controls). This study population (n=1986) constitute the OLIN COPD study. In the current study, for each case and control in the OLIN COPD study, we retrospectively identified data from their first clinical examination, which for cohorts I-III was carried out during the 1980s or 90s. In total, we could include 902 cases and 819 controls in the current study.
